# A long road ahead. A German national survey study on awareness and willingness of surgeons towards the carbon footprint of modern surgical procedures

**DOI:** 10.1016/j.heliyon.2024.e25198

**Published:** 2024-01-24

**Authors:** Sven Jacob, Sophie Anne Schust, Martin Angele, Jens Werner, Markus Guba, Nikolaus Börner

**Affiliations:** Ludwig-Maximilians-University, Department of General, Visceral and Transplantation Surgery, Munich, Germany

## Abstract

**Background:**

Climate change may well be the “largest threat” to humankind. Changes to our climate system lead to a decrease in global health. The healthcare sector presents one of the largest carbon footprints across all industries. Since surgical departments have one of the largest carbon footprints within the healthcare sector, they represent an area with vast opportunities for improvement. To drive change, it is vital to create awareness of these issues and encourage engagement in changes among people working in the healthcare industry.

**Methods:**

We conducted an anonymous cross-sectional survey study to assess awareness among surgeons regarding the impact of healthcare systems on climate change. The questions were designed to investigate surgeons' willingness to accept and promote changes to reduce carbon footprints. Participants included surgical professionals of all ages and levels of expertise.

**Results:**

A total of 210 participants completed the survey in full and were included in the evaluation. Sixty percent emphasized a lack of information and the need for personal education. Over 90 % expressed concern for the environment and a strong desire to gain new insights. Provided that clinical performance remains the same, more than 70 % are willing to embrace carbon-friendly alternatives. In this context, all participants accepted the additional time required for training and initially increased personal efforts to achieve equal performance.

**Conclusion:**

Limited awareness and information about carbon footprints were observed in surgical departments in German hospitals. Nevertheless, the vast majority of surgeons across all age groups are more than willing to acquire new insights and adapt to changes in order to reduce energy consumption and carbon dioxide production.

## Highlights

• The health care system is one of the biggest carbon food prints across all industries, potential for change is undisputable.
• We can observe limited awareness and information on carbon footprints in surgical departments in German hospitals.
• The majority of surgeons is willing to gain new insights and adapt to changes to reduce energy consumption and carbon dioxide production.

## 1 Introduction

Climate change is one of the greatest threats to modern humanity [1,2]. Due to complex changes to our earth's climate, the main driving forces of global health, namely the environment, socioeconomic status and wealth, become even more imbalanced with heavier focus on environmental aspects. As vast areas of land become uninhabitable, there is an even further rise in additional deaths from poverty, malnutrition and displacement [3,4]. As recent years have shown, there is an expected rise in deaths from natural disaster due to global warming. Furthermore, it is evident that climate change is intricately associated with the emergence of novel infectious diseases, and we anticipate a surge in the incidence of epidemics [5].

As demonstrated by Rita et al. during the COVID pandemic, a reduction of our daily activities to a minimum lead to a direct reduction of our carbon dioxide (CO2) footprint and the overall air pollution [6]. Given the World Health Organization's explicit assertion that annually, 3.2 million individuals experience untimely mortality due to ailments directly ascribable to air pollution, it becomes imperative to underscore the potential for substantive reduction in carbon dioxide emissions through alterations in our daily consumption practices [7].

Adding to the strain on the healthcare system, healthcare itself contributes significantly to the problem by generating substantial carbon emissions. These emissions vary, accounting for approximately 5 % in Canada and up to 10 % in the United States (US) [8,9]. Notably, despite comprising only 20 % of the world's population, the US, China, and the European Union collectively shoulder 56 % of the healthcare-related climate footprint, making them the largest contributors in this context [10].

Surgery, in particular, is recognized as one of the primary contributors to carbon emissions within the healthcare sector. This can be attributed, in part, to the substantial energy demand during surgical procedures and the extensive use of medical devices, with a significant portion being disposable.

However, not only the surgical procedure itself but the complete pathway of a patient requiring surgery contributes to the carbon footprint of surgical clinics. As shown in the UK, a net zero surgery is a complex structure involving all contributing units within a patients pathway [11]. Since the greatest potential for CO2 reduction lies in the operations' footprint, we begin our investigation with addressing related issues. Thus, within this survey study, we aim to investigate surgeons' awareness of surgical carbon footprint. Secondly, our goal is to assess the willingness of surgical personnel to embrace changes and actively contribute to achieving a carbon-neutral operating theater. Armed with this understanding, we can facilitate the implementation of experimental studies (as outlined below) aimed at devising effective strategies for driving change. Moreover, the presentation of these findings would be instrumental in gathering support, particularly when endeavoring to secure political and industrial backing for the adoption of revised strategies aimed at mitigating environmental footprints. This may encompass augmenting maintenance budgets or soliciting funding for research proposals.

## 2 Methods

### 2.1 Study design and objectives

Given that the healthcare system is responsible for one of the largest carbon footprints across all industries, the primary objective of this study was to examine surgeons' awareness of CO2 emissions within a perioperative setting. Further, our goal is to highlight surgeons' willingness toward participation in carbon footprint reduction. Therefore, we conducted a voluntary and non-incentivised anonymous cross-sectional open survey of surgeons as well as people working in surgical departments such as surgical assistants in Germany. The population was chosen randomly by reaching out to hospitals in every major city in Germany, aiming to represent a complete picture of the national opinion. Distribution of the survey was done via email invitation including the link to the survey. Primary contact was made with each department chief with the request to share the link in their departments via a group email account of their departments. The email included the link to the survey as well as an abstract on the intent. The survey was kept open for 12 weeks. Monthly reminder emails were sent during the survey period.

**Fig. 1:**
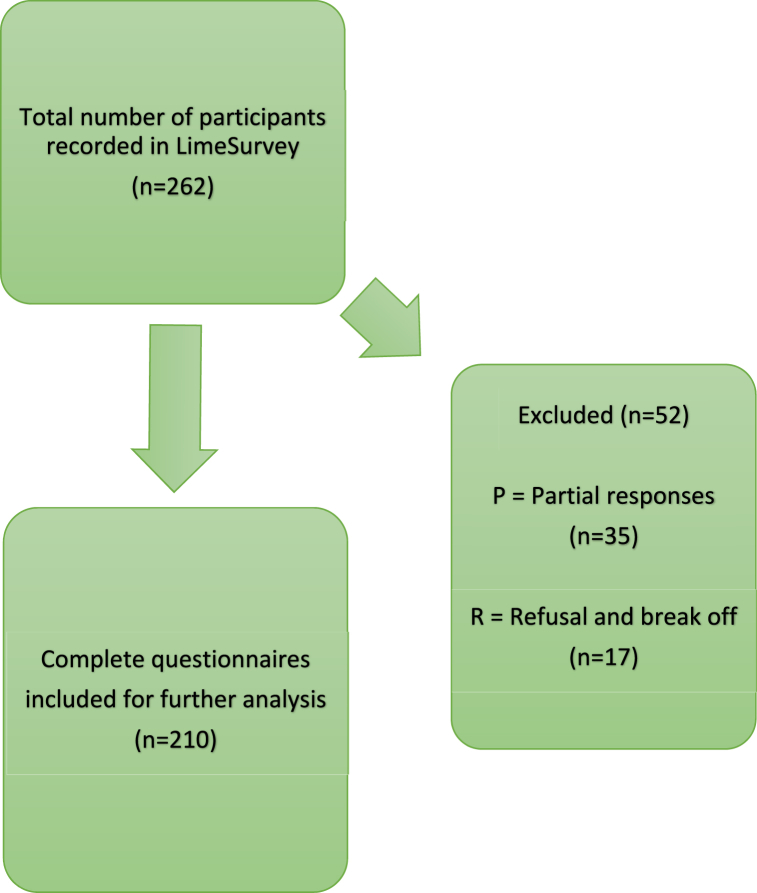
Flow-chart of final survey inclusion procedure.

The survey was produced in Limesurvey (Limesurvey GmbH. LimeSurvey: An Open Source survey tool/LimeSurvey GmbH, Hamburg, Germany. URL http://www.limesurvey.org) designed as a web based fully anonymous survey composed of 46 items and 4 screens. The final survey was structured into four sections. Section A focussed on gathering personal information of each participant, e.g., age and sex. Section B was captured general personal opinions towards climate change, carbon footprints and CO2 consumption in daily life and at the workplace. In section C, questions were designed to address specific aspects in the daily routine of a surgeon, such as the use of specific instruments and reusability, information on product life cycle assessments and packaging [12]. In section D, we aimed to elucidate personal perception of existing barriers towards more CO2 neutral alternatives. To ensure clarity, length, answerability and functioning, the survey was pilot tested at the Ludwig-Maximilian university hospital Munich (n = 20), the results were included in the final analysis. The median time to complete the survey was 6 min, still, longer periods were accepted. Participants had the option to take the survey in German and English. Participants were only able to complete the survey once. In the final analysis, only fully completed surveys were included (See Flow Chart, Fig. 1.). No personal information in terms of names was collected and informed consent was given by taking part in the study which was outlined prior to first question. The survey is in line with The American Association for Public Opinion Research reporting guidelines of Survey Studies (AAPOR) (Guidelines, Definitions Report, Response rate and eligibility rate are lined out in Appendix 1.) and CHERRIES was respected for clarity [13,14].

### 2.2 Statistical analysis

All analyses were performed using SPSS28.0 and GraphPad Prism Version 9.4.0. Answer frequency distributions were calculated and shown in percentages. Univariate and subgroup analysis were done by using chi-squared test for categorial parameters (e.g., different answers between age groups). P values of less than 0.05 were considered significant.

### 2.3 Ethics

The study was presented to the local ethics board and the directing officer of data protection. As it was an anonymous questionnaire, the ethical approval was waived by the ethics Committee of the Ludwig-Maximilian University (LMU) Munich (Survey application ID 1896, process chair Gerhard Meyer, LMU Munich, Pettenkoferstr. 8a, 80336 München, Germany). Every participant was informed that by submitting the questionnaire, consent to the study and its proceedings was given.

## 3 Results

### 3.1 Participants

**Fig. 2:**
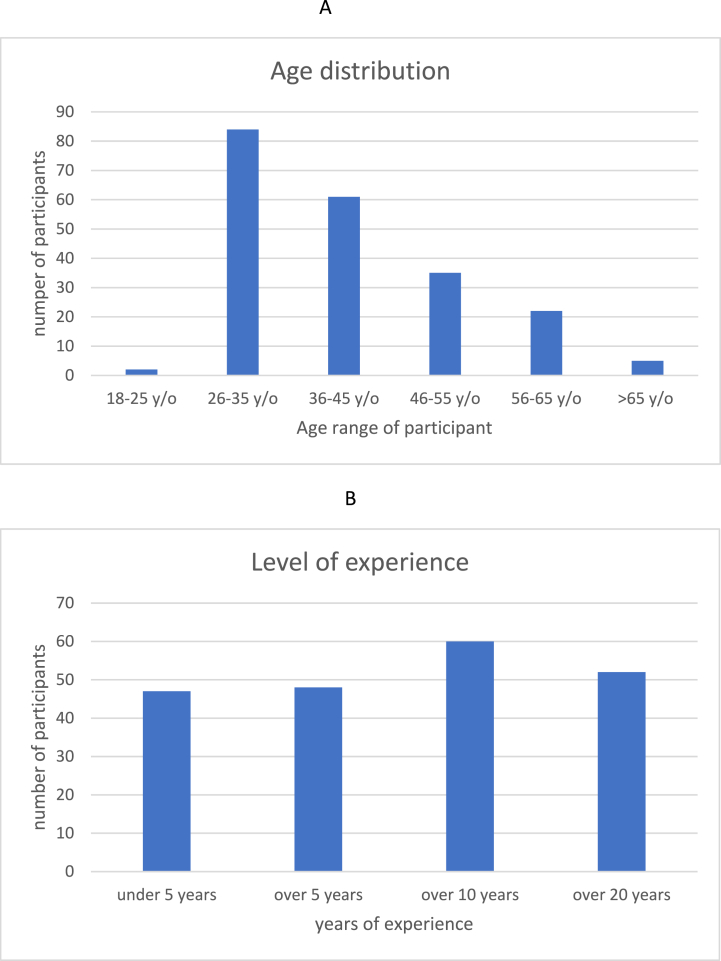
A. Distribution of age. Fig. 2 B. Level of experience.

The survey was sent on April 27, 2022 to 120 chief physicians and other staff of surgical clinics of different care levels, with the request to share the survey within their staff. The survey was kept open for 12 weeks. Monthly reminder emails were sent during the survey period. The survey was initially answered by 262 respondents, 210 of whom fully completed the questionnaire and were included in the analysis. In total, 52 questionnaires were excluded, 35 participants did not answer the complete questionnaires and 17 participants started but did not finish the questionnaire. The survey encompassed 42 hospitals in 35 German cities. Sex distribution was female n = 77 (36.67 %) and male n = 133 (63.33 %). Age and expertise distribution are represented in Fig. 2A and B. The participants included in the evaluation ranged from abdominal surgeons n = 90 (42.86 %), trauma surgeons n = 32 (15.24 %), gynaecologists n = 25 (11.90 %), urologists n = 23 (10.95 %) and others n = 40 (19.05 %, incl. ear-nose throat, neurosurgery, plastic surgery, maxillofacial surgery). Most of the participants are currently working in a primary care hospital n = 162 (77.14 %), n = 32 (15.24 %) in a secondary care hospital and n = 16 (7.62 %) in a tertiary care hospital. * The basic study population characteristics are summarized in Table 1.

**Table 1:** Study population.

Sex		
Male	133	63.33 %
Female	77	36.67 %
Non – binary	0	0 %
**Age**		
18-25	2	0.95 %
26-35	84	40.00 %
36-45	61	29.05 %
46-55	35	16.67 %
56-65	22	10.48 %
>65	6	2.86 %
**Job description**		
Trauma surgeon	32	15.24 %
Abdominal surgeon	90	42.86 %
Urologist	23	10.95 %
Gynaecologist	25	11.90 %
Others	40	12.38 %
**Level of expertise (in years)**		
Under 5 years	47	22.38 %
Over 5 years	48	22.86 %
Over 10 years	60	28.57 %
Over 20 years	52	26.19 %
**Current hospital**		
Specialized hospital with primary care (e.g., university hospital)	162	77.14 %
Regional hospital with secondary care	32	15.24 %
Clinic with tertiary care	15	7.62 %

*Primary care hospitals provide maximum care e.g. university hospitals; secondary care hospitals offer broad medical services and may admit patients referred by tertial care providers but lacking specialties such as neuro surgery; tertiary care hospitals provide basic medical care.

### 3.2 General attitude towards the environment

In total, 92.86 % of participating surgeons state that they “care about the environment in general”. Furthermore, 91.42 % would like to have “more information about environmentally friendlier alternatives to existing products in general”. Another 91.43 % of the participants expect changes in the cost profile when engaging in procedures that reduce C02 consumption. In that context, 63.33 % would accept additional cost if an environmentally friendly alternative would exist. Also, 75.71 % of the participants believe the state has the duty to regulate the reduction of CO2 emissions.

### 3.3 Within the operating theatre in general

Approximately 69 % would hope for more communication of and information about the carbon footprint of surgical interventions and 85.24 % lack a contact person within their operating theatre, who can be addressed about environmental impacts of the operating theatre. Likewise, 66.19 % of the survey participants state that they lack information regarding the environmental impact of the operating theatre to be able to make informed decisions. More than one third (33.81 %) believe that an environmentally friendly operating room (OR) would have a positive effect on the outcome, whereas 45.24 % do not believe in a more positive outcome for the patient. However, 80.47 % also believe that there would be no negative influence on patient outcome.

Furthermore, 56.66 % think that a more environmentally friendly OR would have a positive effect on the ORs efficiency, whereas only 20.48 % disagree and did not believe in a more efficient OR.

**Fig. 3:**
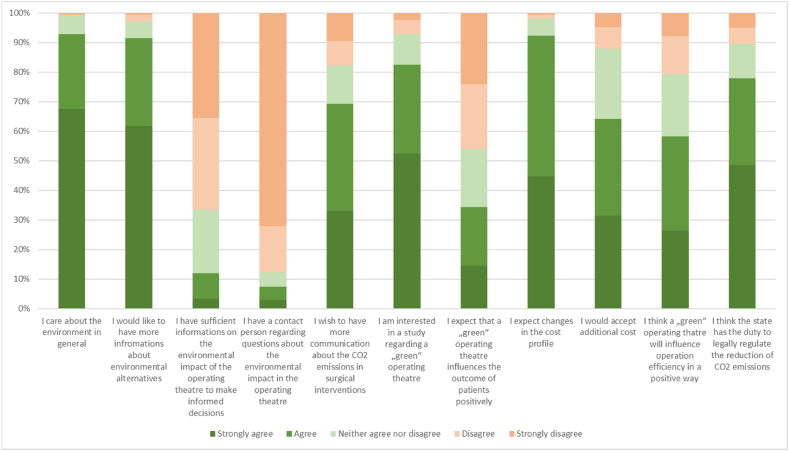
Answer summary regarding attitudes within the operating theatre.

Within the survey, 90.95 % of the participants claimed they would “change their behaviour in the OR if thereby a positive environmental impact” could be achieved. Furthermore, 82.38 % would be highly interested in participating in a “green” and thus CO2-nutral operating theatre study. Answers have been summarized in Fig. 3.

### 3.4 Focus on materials and habits

Within the survey, 71.43 % of respondents consider the supply chain of their instruments, including working conditions in the factories and logistics, as very or somewhat important and 64.34 % want to be actively involved in the process of purchasing the instruments. Furthermore, 78.1 % indicate that their choice of instrument would be influenced by their knowledge of its life cycle assessment. Another 85.71 % state they would prefer the environmentally friendly alternative and 92.38 % even say that the reusability of an instrument is regarded as very important. This goes in line with 80 % of the participants who prefer to use reusable instruments or would even be willing to work with less instruments (84.76 %). Regardless of the fact that 67.14 % claim their “favourite” instrument is regarded as very important, 85 % would be willing to change it if an environmentally friendly alternative exists.

We also asked the surgeons which were there most common barriers towards carbon footprint reduction in the operating theatre. The lack of information about CO2 emissions in the OR is the most crucial reason for 90.48 %. Further, 80.48 % claimed to have to little time to be informed about the subject.

Secondly, 78.57 % stated a lack of guidance for implementing environmentally friendly alternatives is responsible for little change, whereas 78.10 % claim a complete lack of environmentally friendly alternatives exist. There appears to be a great amount of interest for the field as 72.86 % would like their OR management to inform them and 61.43 % would even appreciate regular meetings to discuss improvements. Only 0.95 % of the participants described no barriers to overcome.

### 3.5 Subgroup analysis

In a Subgroup Analysis we compared whether age difference, level of expertise or level of clinic had a significant impact on the answers. There were no statistically significant differences between mentioned groups. Main answers are summarized in Appendix Table 2.

## 4 Discussion

The impact of climate change on public health is becoming increasingly pervasive [15]. As countries increasingly recognize the healthcare system as a significant contributor to climate-related emissions, addressing this issue from a systemic perspective is gaining prominence. More and more nations are taking steps toward achieving climate-neutral healthcare [16,17]. Surgery in particular is considered one of the main emitters of greenhouse gases. It is estimated that the carbon footprint of operating rooms is 3–6 times higher than that of the rest of the hospital's facilities [18,19]. It therefore makes sense to start in surgery where the greatest leverage for CO2 reduction lies. Since the US, Asia and the European Union (EU) present the main emitters, we should feel obliged to take the lead.

Effecting a shift towards climate-neutral operating rooms hinges on raising awareness of the issue and fostering a willingness to alter existing processes. Additionally, it is imperative that the necessary technical capabilities are in place to implement these changes while upholding patient safety as a fundamental premise. As a starting point, this survey was thus designed to address surgeon's awareness of the problem and their willingness to change.

Consistent with findings from other surveys on this topic, our study reveals that over 90 % of respondents share a deep concern for the environment and are open to embracing changes for the sake of enhanced climate protection [20]. Notably, these sentiments are consistent across different age groups and positions within the surgical hierarchy. Intriguingly, a common theme among participants was a perceived lack of information regarding CO2 emissions associated with surgical practices.

In addition, many of the participants felt that they were poorly informed about alternatives and wished to be provided with relevant information to adapt accordingly. This information gap was previously described by Hathaway in 2018, yet the information situation does not appear to have improved significantly since then [21]. One of the factors contributing to this relatively slow change may be the robust regulatory framework in Germany for novel digitalization tools and enhanced information accessibility in hospital settings [22].

Furthermore, addressing climate change requires not only additional financial investment but also an initial time investment, as new procedures need to be implemented. As shown before by, for example, the Health Facilities Management (HFM) and Harris et al., 2017, two of the main barriers to overcome environmental sustainability practices were higher costs over traditional materials and the simultaneous underfunded maintenance budgets [23,24]. While this might still be true for certain areas in the medical supply chain, it is surely not the case in general anymore. In anaesthesia, for example, costs could even be reduced by changing to more environmentally friendly, reusable materials. In fact, it could be shown that cost mitigation potential by changing to reusable devices could be examined in almost all areas. Notably, invasive, e.g., surgical devices, have the lowest potential to reduce costs [25,26]. Within our survey, 84.67 % of the participants would be willing to reduce the amount of instruments used and 63.33 % would accept additional costs. While it is important to acknowledge the potential gap between survey responses and actual actions, these commitments still require follow-up and monitoring [27]. Notably, the subset of 112 participants, constituting 53.33 % of the total, who possess over a decade of experience in the field of surgery assumes particular significance. This group holds the potential to play a pivotal role in advocating for transformative measures within their respective departments since some among them may already hold positions of influence as chief surgeons or department heads. Also, this group of responders surely has the surgical knowledge to decide which changes do not harm patient outcome, if not improve it. In that context, it seems of equal importance to underline that 40 % of all people taking part in the survey analysis were in the age group of 26–35. While it may seem contradictory to the above-mentioned reasoning, in our opinion, it also bears great potential. Even though we could not exhibit statistically significant differences in attitudes towards climate change between different age groups, we do think that socioeconomic aspects such as age can influence behaviour [28]. In that context, we believe that younger people grow up with more awareness of the importance of climate neutral working environments [29]. Further, it is also our opinion that the upcoming generation of surgeons might be able to adapt more rapidly to changes in workflows and working environment. As such, our colleagues in the UK have shown with the “Green Surgery Challenge”, there is movement towards sustainable operating theatres [7]. Further they have shown that a simple alteration of operating instruments already has an impact on emission [30].

While we could demonstrate a very high acceptance for the need of change regarding the CO2 footprint throughout every age group working around the operating theatre, there are still some limitations to this work. The methodology was not designed for a statistically representative survey elucidating on differences between subgroups such as hospital size or degree of specialty. Rather it was meant to gather opinions among staff in surgical departments for planning further studies and pointing out the general acceptance of change. Further, due to the selection of hospitals, there was a preponderance of survey participants from university hospitals and primary health care providers who might well have a different focus towards the intersection between profitability and environmentally friendly workflows. For further investigation, we also propose to include business educated staff working in the hospital management. At first glance, it may seem that for instance changing to reusable devices or implanting new software for daily planning of operating theatres may increase costs. With thorough investigation however, it becomes clear that total health care costs and costs of facility management may well be reduced. Labib et al., for example, could underline a reduction in costs and CO2 consumption by presenting a revised surgical set for appendectomies [31]. As such, people involved in determining the maintenance budgets will be a promising addition. Moreover, as mentioned above, for a detailed investigation of CO2 emission within a surgical department, one has to keep in mind that the full pathway of a patient requiring surgery has to be included, not only the operating theatre. Finally, after 2 years of COVID-19 pandemic and other problems caused by the Ukraine war, the focus of participants may be somewhat less on environmental issues at present.

## 5 Conclusion

Our survey highlights a significant level of awareness among German surgeons regarding the issue of CO2 emissions from the healthcare system. It also underlines a strong willingness among respondents to actively engage in changes aimed at practicing environmentally sustainable surgery within the operating theatre. However, surgeons have difficulties in getting information about the actual CO2 footprint of the underlying processes. Further, they demand guidance to make informed decisions. However, overall acceptance of surgeons towards a climate-neutral working environment was remarkably high. Notably, this acceptance was equal in every age group. Not only potential decision-makers as chiefs of surgery, probably in the age group over 30, but also younger colleagues answered in the same fashion. As a result, it appears not only critically important but also highly promising to allocate more time and effort towards CO2 reduction in the operating theatre. We therefore propose a prospective trial focusing on rearranging the pathway around the operating room. The aim is to reduce its carbon footprint while maintaining the time needed for and the quality of the surgical procedures. Such a trial is currently strategically planned at the LMU hospital, Munich starting from experimenting with new ways of, for example, illumination and climatization planning of an operating room to interchanging surgical devices, such as scissors. Further, we strongly believe that a CO2 label for medical devices can support decision making a great deal, which was also regarded useful by 75 % of the participants.

## Data availability statement

The data used for this investigation has not been deposited into a commonly available data base. However, since he data was acquired anonymously, it can be made available upon request.

## Ethics and consent

As it was an anonymous questionnaire, the ethical approval was waived by the ethics Committee of the Ludwig-Maximilian University Munich (process chair Gerhard Meyer, LMU Munich, Pettenkoferstr. 8a, 80336 München, Germany). Survey application ID 1896. Every participant was informed that by submitting the questionnaire, consent to the study and its proceedings was given.

## CRediT authorship contribution statement

**Sven Jacob:** Writing – review & editing, Writing – original draft, Project administration, Methodology, Investigation, Formal analysis, Conceptualization. **Sophie Anne Schust:** Project administration, Formal analysis, Data curation. **Martin Angele:** Validation, Methodology, Investigation. **Jens Werner:** Validation, Project administation, Methodology, Writing - review & editing. **Markus Guba:** Project administration, Methodology, Conceptualization. **Nikolaus Börner:** Writing – review & editing, Writing – original draft, Methodology, Investigation, Formal analysis, Data curation, Conceptualization.

## Declaration of competing interest

This work has not been published or accepted for publication, nor is it under consideration at another journal. Moreover, I would like to declare on behalf of the authors that there are no ethical, financial nor other conflicts of interests and that all authors have seen and approved the manuscript.

## 7. Abbreviations

CO2: **Carbon dioxide**
EU: **European Union**
HFM: **Health Facilities Management**
LMU: **Ludwig-Maximilian University**
OR: **Operating room**
AAPOR: **The American Association for Public Opinion Research**
US: **United States**
WHO: **World Health Organization**
