# Supplementary material for: A long road ahead. A German national survey study on awareness and willingness of surgeons towards the carbon footprint of modern surgical procedures
Source: Heliyon. 2024 Jan 24;10(3):e25198. doi: 10.1016/j.heliyon.2024.e25198 (PMC10847866; doi:10.1016/j.heliyon.2024.e25198)
Supplement: Multimedia component 1 [file mmc1.docx]

Appendix 1.

AAPOR Reporting Guidelines

**Eligibility rate of the Survey:**

As we reached out to the chiefs of the departments, we assumed that all respondents were eligible to answer the survey. The chief was specifically addressed by name. He was asked to distribute the survey to his staff. His employees are still working as surgeons at his hospital. Therefore, all recipients are eligible respondents. A lack of a returned questionnaire would henceforth designate that person as a eligible, non-responder.

Within the survey, we asked the participant weather he is an active surgeon to reassure his eligibility.

**Response rate of the Survey:**

Returned questionnaires were divided into two groups: a) complete and b) partial. We defined that 100% of questions had to be answered for a complete response and more than 50% for a partial response. In the final analysis, we only included the complete responses.

Table presenting the response rate calculation by “AAOPOR Calculator 4.1.”

|  |  |
| --- | --- |
| Total sample used | 1182 |
|  |  |
| I=Complete Interviews | 210 |
| P=Partial Interviews | 35 |
| R=Refusal and break off | 17 |
| NC=Non Contact | 600 |
| O=Other | 0 |
| Calculating e, by AAPOR's 2009 Eligibility Estimates. | 1 |
| UH=Unknown Household | 0 |
| UO=Unknown other | 0 |

| **Response Rate 1** |  |
| --- | --- |
| I/((I+P)+(R+NC+O)+(UH+UO)) | 0,244 |
| **Response Rate 2** |  |
| (I+P)/((I+P)+(R+NC+O)+(UH+UO)) | 0,284 |
| **Response Rate 3** |  |
| I/((I+P)+(R+NC+O)+e(UH+UO)) | 0,244 |
|  |  |
| **Response Rate 4** |  |
| (I+P)/((I+P)+(R+NC+O)+e(UH+UO)) | 0,284 |
| **Cooperation Rate 1** |  |
| I/((I+P)+R+O) | 0,802 |
| **Cooperation Rate 2** |  |
| (I+P)/((I+P)+R+O)) | 0,935 |
| **Cooperation Rate 3** |  |
| I/((I+P)+R) | 0,802 |
| **Cooperation Rate 4** |  |
| (I+P)/((I+P)+R) | 0,935 |
| **Refusal Rate 1** |  |
| R/((I+P)+(R+NC+O)+(UH+UO)) | 0,020 |
| **Refusal Rate 2** |  |
| R/((I+P)+(R+NC+O)+e(UH+UO)) | 0,020 |
| **Refusal Rate 3** |  |
| R/((I+P)+(R+NC+O)) | 0,020 |
| **Contact Rate 1** |  |
| ((I+P)+R+O)/((I+P)+(R+NC+O)+(UH+UO)) | 0,304 |
| **Contact Rate 2** |  |
| ((I+P)+R+O)/((I+P)+(R+NC+O)+e(UH+UO)) | 0,304 |
| **Contact Rate 3** |  |
| ((I+P)+R+O)/((I+P)+(R+NC+O)) | 0,304 |
