# Supplementary material for: A long road ahead. A German national survey study on awareness and willingness of surgeons towards the carbon footprint of modern surgical procedures
Source: Heliyon. 2024 Jan 24;10(3):e25198. doi: 10.1016/j.heliyon.2024.e25198 (PMC10847866; doi:10.1016/j.heliyon.2024.e25198)
Supplement: Multimedia component 2 [file mmc2.docx]

Appendix 2. Subgroup Analysis

| Q: I care about the environment in general |
| --- |
| Q: I would like to have more information about environmental alternatives |
| Q: I have sufficient information on the environmental impact of the operating theatre to make informed decisions |
| Q: I have a contact person regarding questions about the environmental impact in the operating theatre |
| Q: I am interested in a study regarding a „green“ operating theatre |
| Q: I am willing to change my behavior in the operating theatre if thereby a positive environmental impact can be achieved |
| Q: I expect changes in the cost profile |
| Q: I would accept additional cost |
| Q: I think the state has the duty to legally regulate the reduction of CO2 emissions |

**Table 1**

*Subgroup Analysis based on Age*

|  | **18-25 y/o** |  |  | **26-35 y/o** |  |  | **36-45 y/o** |  |  | **46-55 y/o** |  |  | **56-65 y/o** |  |  | **>65 y/o** |  |  | **Chi-squared**  **p-value** |
| --- | --- | --- | --- | --- | --- | --- | --- | --- | --- | --- | --- | --- | --- | --- | --- | --- | --- | --- | --- |
|  | **Agreeing %** | **Neutral %** | **Not agreeing %** | **Agreeing %** | **Neutral %** | **Not agreeing %** | **Agreeing %** | **Neutral %** | **Not agreeing %** | **Agreeing %** | **Neutral %** | **Not agreeing %** | **Agreeing %** | **Neutral %** | **Not agreeing %** | **Agreeing %** | **Neutral %** | **Not agreeing %** |  |
| I care about the environment (in general) | 100 | 0 | 0 | 90,5 | 9,5 | 0 | 95,1 | 4,9 | 0 | 94,3 | 5,7 | 0 | 100 | 0 | 0 | 80 | 20 | 0 | 0,47 |
| I would like to have more informations about environmental alternatives | 100 | 0 | 0 | 88,1 | 10,7 | 1,2 | 95,1 | 0 | 4,9 | 94,3 | 2,9 | 2,9 | 86,4 | 9,1 | ,+T4 | 100 | 0 | 0 | 0,363 |
| I have sufficient informations on the environmental impact of the operating theatre to make informed decisions | 50 | 0 | 50 | 10,7 | 15,5 | 73,8 | 8,2 | 19,7 | 70,5 | 14,3 | 22,9 | 62,9 | 22,7 | 40,9 | 36,4 | 0 | 40 | 60 | 0,079 |
| I have a contact person regarding questions about the environmental impact in the operating theatre | 0 | 0 | 100 | 1,6 | 6,6 | 90,2 | 11,4 | 5,7 | 77,1 | 11,4 | 5,7 | 77,1 | 27,3 | 4,5 | 68,2 | 0 | 20 | 60 | 0,011 |
| I am interested in a study for a „green“ operating theatre | 100 | 0 | 0 | 86,9 | 6 | 6 | 82 | 9,8 | 8,2 | 74,3 | 14,3 | 11,4 | 68,2 | 22,7 | 9,1 | 100 | 0 | 0 | 0,779 |
| I am willing to change my behavior in the operating theatre if thereby a positive environmental impact can be achieved | 100 | 0 | 0 | 91,7 | 6 | 2,4 | 93,4 | 1,6 | 4,9 | 82,9 | 8,6 | 2,9 | 90,9 | 4,5 | 4,5 | 100 | 0 | 0 | 0,937 |
| I expect changes in the cost profile | 100 | 0 | 0 | 91,7 | 6 | 2,4 | 91,8 | 4,9 | 1,6 | 91,4 | 2,9 | 2,9 | 90,9 | 9,1 | 0 | 80 | 20 | 0 | 0,956 |
| I think the state has the duty to legally regulate the reduction of CO2 emissions | 100 | 0 | 0 | 20,2 |  | 6 | 13,1 | 1,6 | 1,6 | 17,1 | 2,9 | 0 | 18,2 | 4,5 | 0 | 20 | 0 | 0 | 0,535 |
| *N=210; Agreeing= including „strongly agree“ and „agree, not agreeing= including “disagree” and “strongly not agreeing* | | | | | | | | | | | | | | | | | | | |

**Table 2**

*Subgroup Analysis based on Years of Expertise*

|  | **Under 5 years** |  |  | **Over 5 years** |  |  | **Over 10 years** |  |  | **Over 20 years** |  |  | **Chi-squared**  **p-value** |  |
| --- | --- | --- | --- | --- | --- | --- | --- | --- | --- | --- | --- | --- | --- | --- |
|  | **Agreeing %** | **Neutral %** | **Not agreeing %** | **Agreeing %** | **Neutral %** | **Not agreeing %** | **Agreeing %** | **Neutral %** | **Not agreeing %** | **Agreeing %** | **Neutral %** | **Not agreeing %** |  |  |
| I care about the environment (in general) | 91,5 | 8,5 | 0 | 89,6 | 10,4 | 0 | 95,0 | 5,0 | 0 | 96,2 | 3,8 | 0 | 0,524 |  |
| I would like to have more informations about environmental alternatives | 91,5 | 8,5 | 0 | 87,5 | 10,4 | 2,1 | 95,0 | 0 | 5,0 | 92,3 | 3,8 | 3,8 | 0,154 |  |
| I have sufficient informations on the environmental impact of the operating theatre to make informed decisions | 6,4 | 14,9 | 78,7 | 14,6 | 16,7 | 68,8 | 8,3 | 21,7 | 68,3 | 19,2 | 30,8 | 50,0 | 0,082 |  |
| I have a contact person regarding questions about the environmental impact in the operating theatre | 6,4 | 2,1 | 89,4 | 0 | 2,1 | 93,8 | 5,0 | 6,7 | 88,3 | 17,3 | 7,7 | 69,2 | 0,112 |  |
| I am interested in a study for a „green“ operating theatre | 95,7 | 2,1 | 2,1 | 81,3 | 8,3 | 8,3 | 80,0 | 11,7 | 8,3 | 73,1 | 15,4 | 11,5 | 0,175 |  |
| I am willing to change my behavior in the operating theatre if thereby a positive environmental impact can be achieved | 97,9 | 2,1 | 0 | 87,5 | 8,3 | 4,2 | 93,3 | 1,7 | 5,0 | 84,6 | 7,7 | 3,8 | 0,347 |  |
| I expect changes in the cost profile | 93,6 | 4,3 | 2,1 | 91,7 | 6,3 | 2,1 | 91,7 | 5,0 | 1,7 | 88,5 | 7,7 | 1,9 | 0,995 |  |
| I think the state has the duty to legally regulate the reduction of CO2 emissions | 23,4 | 0 | 6,4 | 14,6 | 0 | 4,2 | 16,7 | 1,7 | 1,7 | 15,4 | 3,8 | 0 | 0,316 |  |
| *N=210; Agreeing= including „strongly agree“ and „agree, not agreeing= including “disagree” and “strongly not agreeing* | | | | | | | | | | | | | | |

**Table 3**

*Subgroup Analysis based on Hospital Size*

|  | **Specialized hospital with maximum care (e.g. university hospital)** | |  |  | **regional hospital with medium care** |  |  | **clinic with basic care** |  |  | **Chi-squared**  **p-value** |
| --- | --- | --- | --- | --- | --- | --- | --- | --- | --- | --- | --- |
|  | **Agreeing %** | **Neutral %** | | **Not agreeing %** | **Agreeing %** | **Neutral %** | **Not agreeing %** | **Agreeing %** | **Neutral %** | **Not agreeing %** |  |
| I care about the environment (in general) | 91,4 | 8,6 | | 0 | 100 | 0 | 0 | 100 | 0 | 0 | 0,113 |
| I would like to have more informations about environmental alternatives | 90,1 | 7,4 | | 2,5 | 93,8 | 0 | 6,3 | 100 | 0 | 0 | 0,245 |
| I have sufficient informations on the environmental impact of the operating theatre to make informed decisions | 11,7 | 21,6 | | 66,0 | 12,5 | 21,9 | 65,6 | 13,3 | 13,3 | 73,3 | 0,962 |
| I have a contact person regarding questions about the environmental impact in the operating theatre | 5,6 | 4,9 | | 85,8 | 9,4 | 3,1 | 87,5 | 20,0 | 6,7 | 73,3 | 0,324 |
| I am interested in a study for a „green“ operating theatre | 80,2 | 10,5 | | 8,6 | 90,6 | 6,3 | 3,1 | 86,7 | 6,7 | 6,7 | 0,879 |
| I am willing to change my behavior in the operating theatre if thereby a positive environmental impact can be achieved | 90,1 | 4,3 | | 4,3 | 93,8 | 6,3 | 0 | 93,3 | 6,7 | 0 | 0,665 |
| I expect changes in the cost profile | 90,1 | 6,8 | | 2,5 | 93,8 | 3,1 | 0 | 100 | 0 | 0 | 0,579 |
| I think the state has the duty to legally regulate the reduction of CO2 emissions | 17,3 | 1,9 | | 3,7 | 18,8 | 0 | 0 | 13,3 | 0 | 0 | 0,657 |
| *N=210; Agreeing= including „strongly agree“ and „agree, not agreeing= including “disagree” and “strongly not agreeing* | | | | | | | | | | | |
