# Supplementary material for: A long road ahead. A German national survey study on awareness and willingness of surgeons towards the carbon footprint of modern surgical procedures
Source: Heliyon. 2024 Jan 24;10(3):e25198. doi: 10.1016/j.heliyon.2024.e25198 (PMC10847866; doi:10.1016/j.heliyon.2024.e25198)
Supplement: Multimedia component 3 [file mmc3.pdf]

LimeSurvey

Section A: Personal information

A1. **Please state your sex**

This is a question help text.

- male ☐
- female ☐
- divers ☐

A2. **Please state your age**

- 18-25 y/o ☐
- 26-35 y/o ☐
- 36-45 y/o ☐
- 46-55 y/o ☐
- 56-65 y/o ☐
- >65 y/o ☐

A3. **Please state your country**

A4. **Please state your city**

A5. Please state your job description

- trauma surgeon ☐
- abdominal surgeon ☐
- urologist ☐
- gynecologist ☐
- surgeon other specialization ☐
- scrub nurse ☐
- student ☐
- Other ☐

Other

A6. Please state your level of expertise (years of work experience)

- under 5 years ☐
- over 5 years ☐
- over 10 years ☐
- over 20 years ☐

A7. Please state your current hospital

- specialized hospital with maximum care (e.g. university hospital) ☐
- regional hospital with medium care ☐
- clinic with basic care ☐

Section B: Opening statements

Please mark the following statements depending on how important they are to you.

B1. Please mark the following statements depending on how important they are to you.

	strongly agree	agree	neither agree nor disagree	disagree	strongly disagree
I care about the environment (in general)	☐	☐	☐	☐	☐
I would like to have more informations about environmental alternatives	☐	☐	☐	☐	☐
I have sufficient informations on the environmental impact of the operating theatre to make informed decisions	☐	☐	☐	☐	☐
I have a contact person regarding questions about the environmental impact in the operating theatre	☐	☐	☐	☐	☐

LimeSurvey

	strongly agree	agree	neither agree nor disagree	disagree	strongly disagree
I wish to have more communication about the CO_2 emissions of surgical interventions	☐	☐	☐	☐	☐
I am interested in a study for a „green“ operating theatre	☐	☐	☐	☐	☐
I expect that a „green“ operating theatre influences the outcome of patients positively	☐	☐	☐	☐	☐
I expect that a „green“ operating theatre influences the outcome of patients negatively	☐	☐	☐	☐	☐
I expect that a „green“ operating theatre does not influence the outcome of patients	☐	☐	☐	☐	☐
I am willing to change my behavior in the operating theatre if thereby a positive environmental impact can be achieved	☐	☐	☐	☐	☐
I expect changes in the cost profile	☐	☐	☐	☐	☐
I would accept additional cost	☐	☐	☐	☐	☐
I think a "green" operating theatre will influence operation efficiency in a positive way	☐	☐	☐	☐	☐
I think a "green" operating theatre will influence operation efficiency in a negative way	☐	☐	☐	☐	☐
I think a "green" operating theatre will influence operation efficiency in no way	☐	☐	☐	☐	☐
I think the state has the duty to legally regulate the reduction of CO_2 emissions	☐	☐	☐	☐	☐

Section C: Instrumental questions

Please assess in the next section how important the following factors are for you regarding the choice of instruments

C1.

	very important/ strongly agree	somewhat important/ agree	neutral	somewhat unimportant/ disagree	not important/ strongly disagree
How important are factors relating to the supply chain (working conditions in factories, logistics)?	☐	☐	☐	☐	☐
Would the knowledge about the CO_2 footprint of your surgical device influence your choice?	☐	☐	☐	☐	☐
How important is the cost when choosing your surgical device?	☐	☐	☐	☐	☐
How important is the potential to reuse or recycle a device when choosing your surgical instrument?	☐	☐	☐	☐	☐
How important is the material and and quantity of the packing?	☐	☐	☐	☐	☐
How important is your „favorite“ instrument to you?	☐	☐	☐	☐	☐
Would you consider choosing a qualitatively equivalent alternative if this can improve the environmental impact?	☐	☐	☐	☐	☐
Would the knowledge about the CO_2 footprint of your surgical device influence your choice?	☐	☐	☐	☐	☐
Would you be willing to work with fewer instruments or rather only opening/using items when required?	☐	☐	☐	☐	☐

	very important/ strongly agree	somewhat important/ agree	neutral	somewhat unimportant/ disagree	not important/ strongly disagree
I prefer medical disposables over reusables	☐	☐	☐	☐	☐
I prefer reusables over disposables	☐	☐	☐	☐	☐
How important is it for you to actively be involved in the purchasing of instruments?	☐	☐	☐	☐	☐
Would a sustainability-score (similar to a nutri-score) influence your choice? (A/green= low-emission; E/red=not low-emission)	☐	☐	☐	☐	☐

Section D: Barriers

Which of the following factors are barriers to implement „green“ surgery initiatives in your hospital? (Please tick as appropriate. Multiple replies are possible)

D1.

- Lack of informations about CO2 emissions in the operating theatre ☐
- Lack of guidance for implementing environmental friendly alternatives ☐
- Lack of support from the operating theatre management to improve CO2 emissions ☐
- Lack of environmental friendly choices (e.g. choice of instruments) ☐
- Lack of time to familiarise with the subject ☐
- Lack of regulary meetings to think about suggestions for improvements (e.g. are there suggestions for improvement about current used trays) ☐
- No barriers ☐
- Other ☐

Other

[ ]

Thank you very much for participating in this survey!

We hope you liked the questions and it was thought-provoking.

If you have further questions, don't hesitate to get in contact with us.
